# Targeting Cancer Metabolism as a New Strategy to Enhance Treatment Efficacy and Overcome Resistance

**DOI:** 10.3390/cancers16213629

**Published:** 2024-10-28

**Authors:** Paola Tucci

**Affiliations:** Department of Pharmacy, Health and Nutritional Sciences, University of Calabria, 87036 Rende, CS, Italy; paola.tucci@unical.it; Tel.: +39-0984493185

The intricate relationship between metabolism and cancer has been a subject of growing interest in recent years, as metabolic reprogramming is recognized as one of the hallmarks of cancer [1,2,3]. Tumor cells exhibit profound alterations in their metabolic pathways that influence many, if not all, facets of tumor biology, including the growth, proliferation, and invasion of cancer cells as well as treatment resistance and metastasis [4,5,6,7,8]. These metabolic changes can be caused by mutations or modifications in the cancer cells, but the patient’s metabolism also plays an important role. These metabolic alterations create unique vulnerabilities, which can be exploited for the development of new antitumoral strategies [9,10,11,12].

To this end, this Special Issue of *Cancers* brings together cutting-edge research on the metabolic alterations that occur in cancer, offering insights into novel therapeutic strategies targeting metabolic pathways to deprive cancer cells of the biochemical resources they have come to depend on.

This Special Issue opens with an article by Banella et al., which introduces a promising new approach for the treatment of Acute Myeloid Leukemia (AML). AML is a highly aggressive cancer with a poor prognosis, particularly in elderly patients who are often unfit for intensive chemotherapy. The study investigates the combination of ascorbate (vitamin C) with buformin, a biguanide that inhibits mitochondrial complex I. This combination leverages the metabolic peculiarities of AML cells, which are known to exhibit highly flexible and aggressive metabolic phenotypes. Ascorbate induces oxidative stress by interfering with the glycolytic pathway, while buformin blocks mitochondrial ATP production, effectively starving the cancer cells of energy. The study shows that this combination has a synergistic effect, significantly enhancing apoptosis in AML cells, particularly in primary blasts from elderly patients who are resistant to other treatments. Thus, this metabolic-targeted therapy offers a potential new avenue for treating a difficult-to-treat population.

Following this, Di Magno et al. present an in-depth review of biguanides, focusing on their mechanisms of action in cancer beyond their well-known inhibition of mitochondrial complex I. Biguanides such as metformin and buformin have garnered attention for their antitumor properties, which are believed to stem from their ability to induce energy stress in cancer cells. However, the review highlights that the concentrations of biguanides needed to inhibit complex I in vivo are much higher than what can be achieved in patients without toxicity. This discrepancy has led researchers to explore alternative mechanisms by which biguanides exert their effects. Di Magno and colleagues discuss emerging evidence suggesting that biguanides may affect other metabolic pathways, including redox balance, AMPK activation, and interference with the tumor microenvironment. They also examine the ongoing clinical trials investigating the use of biguanides in cancer therapy, emphasizing the importance of understanding the precise molecular targets of these drugs to maximize their therapeutic potential.

Li et al. take the exploration of metabolic pathways in a different direction, focusing on cholesterol metabolism. Their study investigates the role of SMG1, a kinase involved in nonsense-mediated RNA decay, in regulating cholesterol homeostasis through p53 alternative splicing. p53, a key tumor suppressor, has multiple isoforms, including p53β and p53γ, which have distinct roles in cancer metabolism. Li et al. demonstrate that inhibition of SMG1 increases the expression of these p53 isoforms, leading to altered cholesterol metabolism in cancer cells. This study is particularly important because cholesterol metabolism has been implicated in cancer progression, especially in aggressive tumors that rely on lipid biosynthesis for growth. By uncovering the link between SMG1, p53 isoforms, and cholesterol metabolism, the authors provide new insights into how metabolic reprogramming can be targeted in cancer therapy.

In the context of glycolysis, Zheng et al. present a pan-cancer analysis of the gene SLC2A1, which encodes the glucose transporter GLUT1. Enhanced glycolysis, also known as the Warburg effect, is a hallmark of cancer metabolism, and SLC2A1 plays a central role in this process by facilitating glucose uptake into cancer cells. Zheng et al. show that SLC2A1 is overexpressed in a wide range of cancers and is associated with poor prognosis. The study also highlights the role of SLC2A1 in modulating the tumor microenvironment, particularly in relation to immune evasion. SLC2A1 expression was found to correlate with biomarkers of T-cell exhaustion, such as PD-L1 and CTLA4, suggesting that it may play a role in suppressing the immune response against tumors. This finding positions SLC2A1 not only as a potential therapeutic target, but also as a biomarker for selecting patients who might benefit from immunotherapy.

Hypoxia is another critical aspect of the tumor microenvironment that drives metabolic reprogramming. In their study, Ramírez-Tortosa et al. investigate the role of Hypoxia-Inducible Factor-1 alpha (HIF-1α) in predicting the response to neoadjuvant chemotherapy in breast cancer. HIF-1α is a transcription factor that is stabilized under low oxygen conditions and regulates the expression of genes involved in glycolysis, angiogenesis, and survival under hypoxic conditions. The authors found that HIF-1α expression correlates with a higher likelihood of achieving a pathological complete response (pCR) following chemotherapy. Additionally, HIF-1α was associated with more aggressive tumor features, such as higher Ki-67 levels and hormone receptor negativity. These findings suggest HIF-1α could as a valuable biomarker for predicting treatment response and tailoring therapy in breast cancer patients.

Ragni et al. take a broader view of cancer metabolism, focusing on amino acids and their role in both tumor growth and cancer-associated cachexia. Cancer cells rely on amino acids not only for protein synthesis but also for energy production and signaling. The review discusses the potential of targeting amino acid metabolism in cancer therapy, either by depriving cancer cells of essential amino acids or by supplementing amino acids to support the host’s metabolism in cases of cachexia. Cachexia is a debilitating syndrome characterized by extreme weight loss and muscle wasting, often seen in advanced cancer patients. Ragni et al. argue that while amino acid deprivation can be effective in slowing tumor growth, it may exacerbate cachexia, highlighting the need for a delicate balance in designing therapeutic strategies that consider both the tumor and the host.

Cuyàs et al. explore the metabolic changes associated with the epithelial-to-mesenchymal transition (EMT), a process that plays a key role in cancer metastasis and therapy resistance. Using breast cancer cells, the authors profile the metabolic and mitochondrial alterations that occur during EMT. They find that EMT is associated with increased mitochondrial utilization of glycolytic end-products, as well as a shift towards oxidative metabolism. These changes make EMT cells more resistant to mitochondrial inhibitors, which could explain why EMT-activated cancer cells are more resistant to conventional therapies. This study provides important insights into how targeting metabolic vulnerabilities in EMT cells could enhance the effectiveness of cancer treatments.

The role of obesity in cancer progression is highlighted in a novel study by Iftikhar et al., who examine how epiploic adipose tissue (EPAT) promotes colorectal cancer (CRC) in obese individuals. EPAT is a visceral fat deposit attached to the colon, and the authors show that in obese patients, EPAT creates a tumor-promoting microenvironment that enhances the migration and growth of colon cancer cells. Using a novel microphysiological system, they demonstrate that EPAT from obese individuals releases factors that drive cancer progression, linking obesity with increased CRC risk. This study opens up new avenues for exploring how targeting EPAT could mitigate obesity-associated cancer risks.

Daverio et al. review the phenomenon of lactic acidosis in the tumor microenvironment and how it helps cancer cells resist glucose deprivation. Lactic acidosis arises from the Warburg effect, where cancer cells produce lactate even in the presence of oxygen. The review discusses how lactic acidosis rewires cancer metabolism, promoting a switch from glycolysis to oxidative metabolism, allowing cancer cells to survive in glucose-limited environments. This metabolic flexibility is a key reason why tumors can resist therapies that target glycolysis, and the authors suggest lactic acidosis itself as a promising therapeutic target.

Finally, Tambay et al. use metabolomics to identify a distinctive metabolic signature for hepatocellular carcinoma (HCC). By comparing HCC tissue with adjacent non-tumoral liver tissue, they identify specific metabolites that are altered in cancer, including changes in glutathione, succinate, and alanine levels. These findings support the concept of metabolic reprogramming in HCC and highlight potential biomarkers for early detection and targets for therapy.

In conclusion, the articles (six research, one communication, and one review paper) collected in this Special Issue of *Cancers* highlight the central role of metabolism in cancer biology. The studies discussed here not only underline the impact of changes in metabolism in promoting the spread of cancer, but also create new avenues for targeted treatments that take advantage of the particular metabolic weaknesses in cancer cells. As the field of cancer metabolism continues to evolve, it holds great promise for improving patient outcomes through more effective and personalized treatment strategies.
